# Supplementary figures and images for: The bacterial quorum sensing signal 2’-aminoacetophenone rewires immune cell bioenergetics through the Ppargc1a/Esrra axis to mediate tolerance to infection
Source: eLife. 2024 Sep 13;13:RP97568. doi: 10.7554/eLife.97568 (PMC11398867; doi:10.7554/eLife.97568)

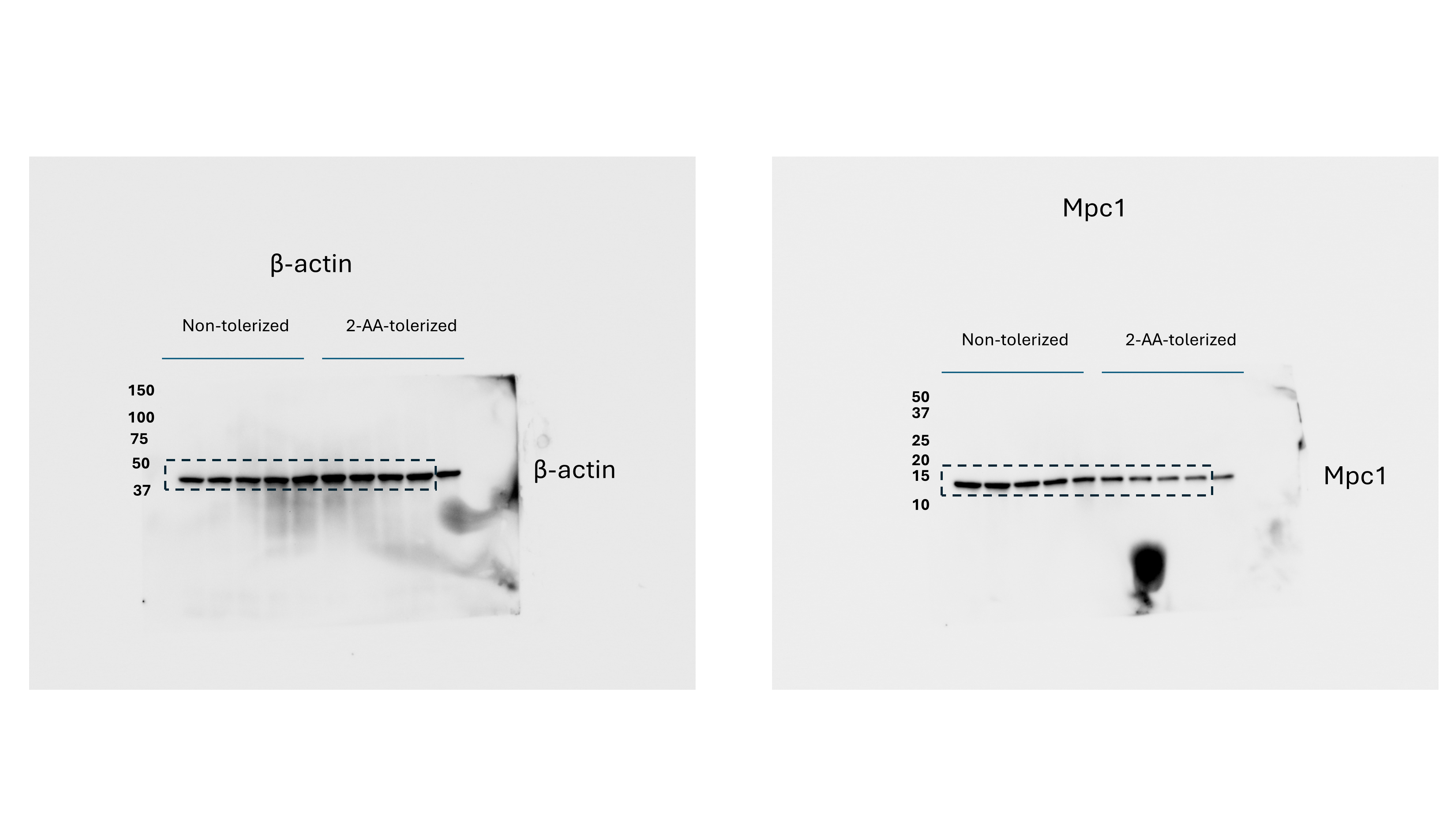

Supplement: Figure 2—source data 2. [file elife-97568-fig2-data2.zip › Figure 2—source data 1.tif]

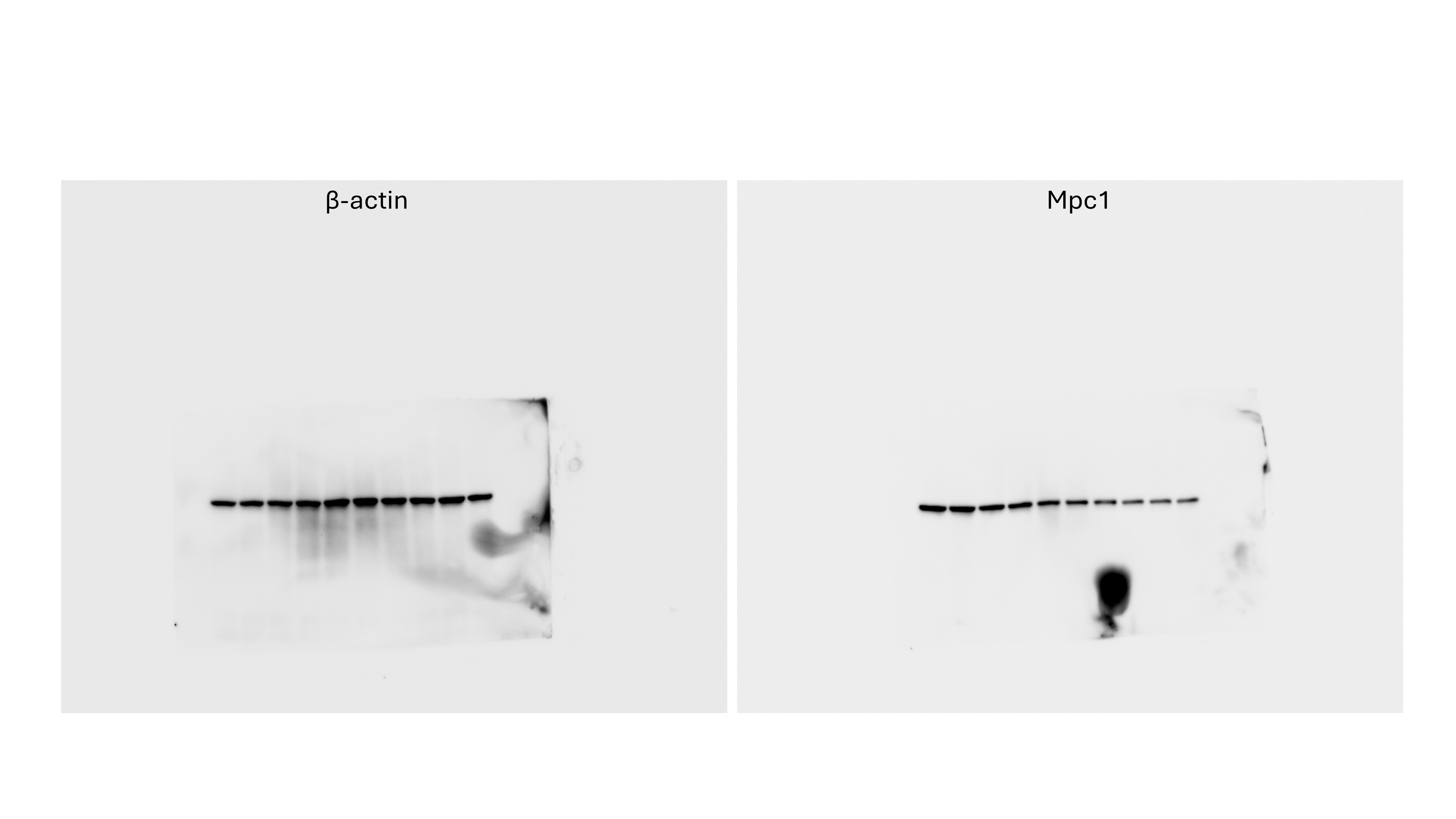

Supplement: Figure 2—source data 3. [file elife-97568-fig2-data3.zip › Figure 2—source data 2.tif]

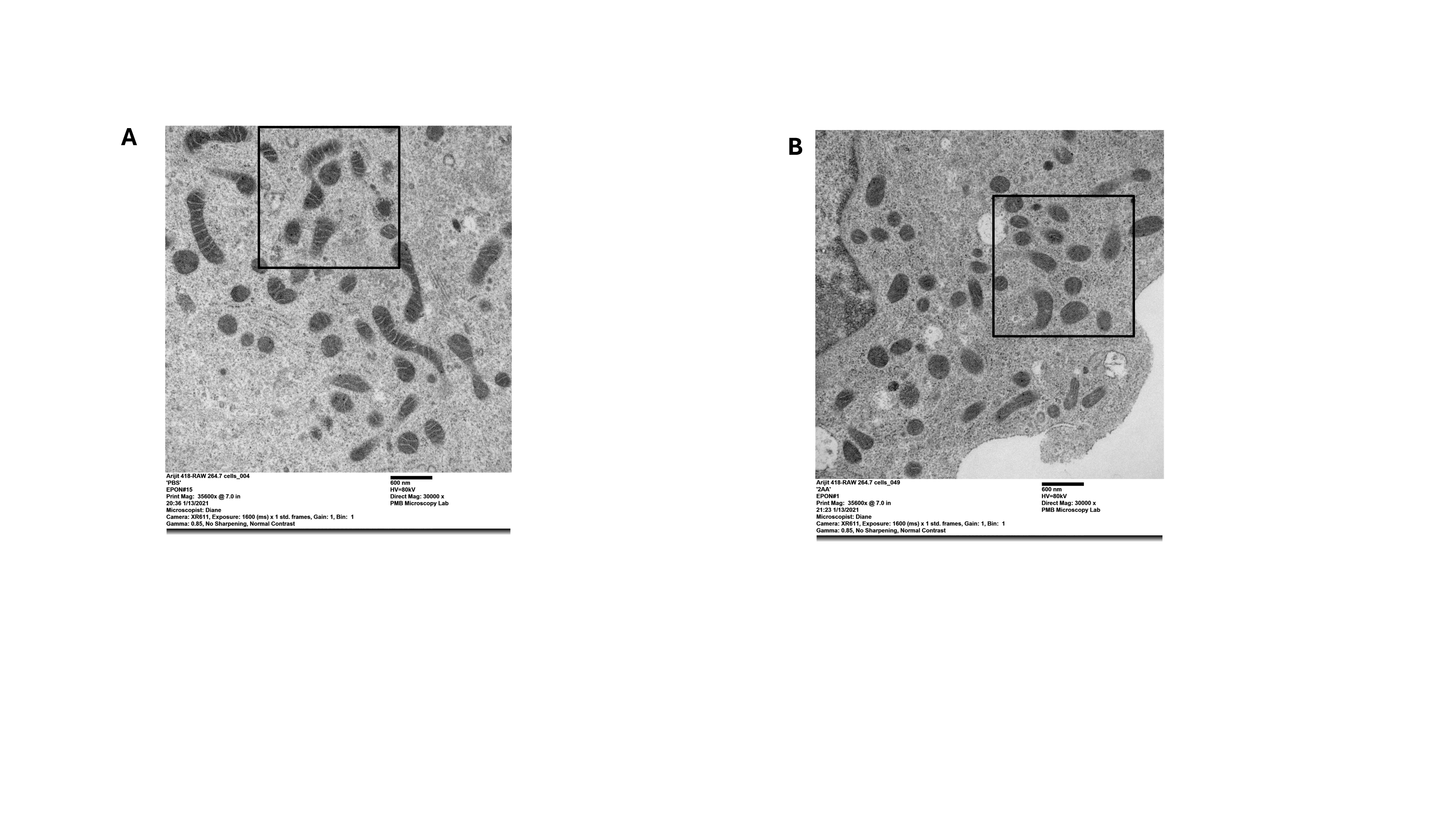

Supplement: Figure 2—figure supplement 1—source data 3. [file elife-97568-fig2-figsupp1-data3.zip › Figure 2-figure supplement 1-source data 1.tif]

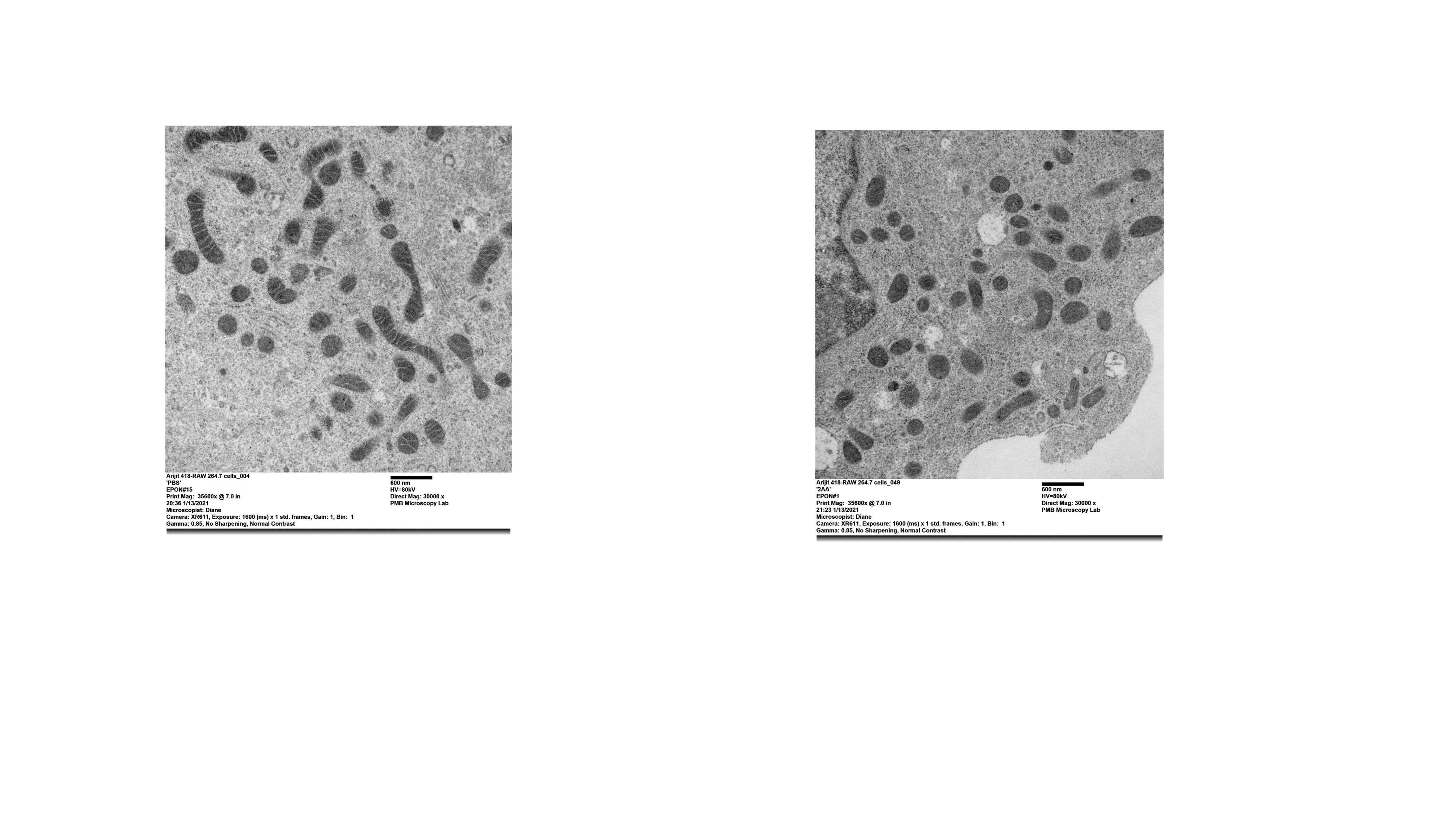

Supplement: Figure 2—figure supplement 1—source data 4. [file elife-97568-fig2-figsupp1-data4.zip › Figure 2-figure supplement 1-source data 2.tif]

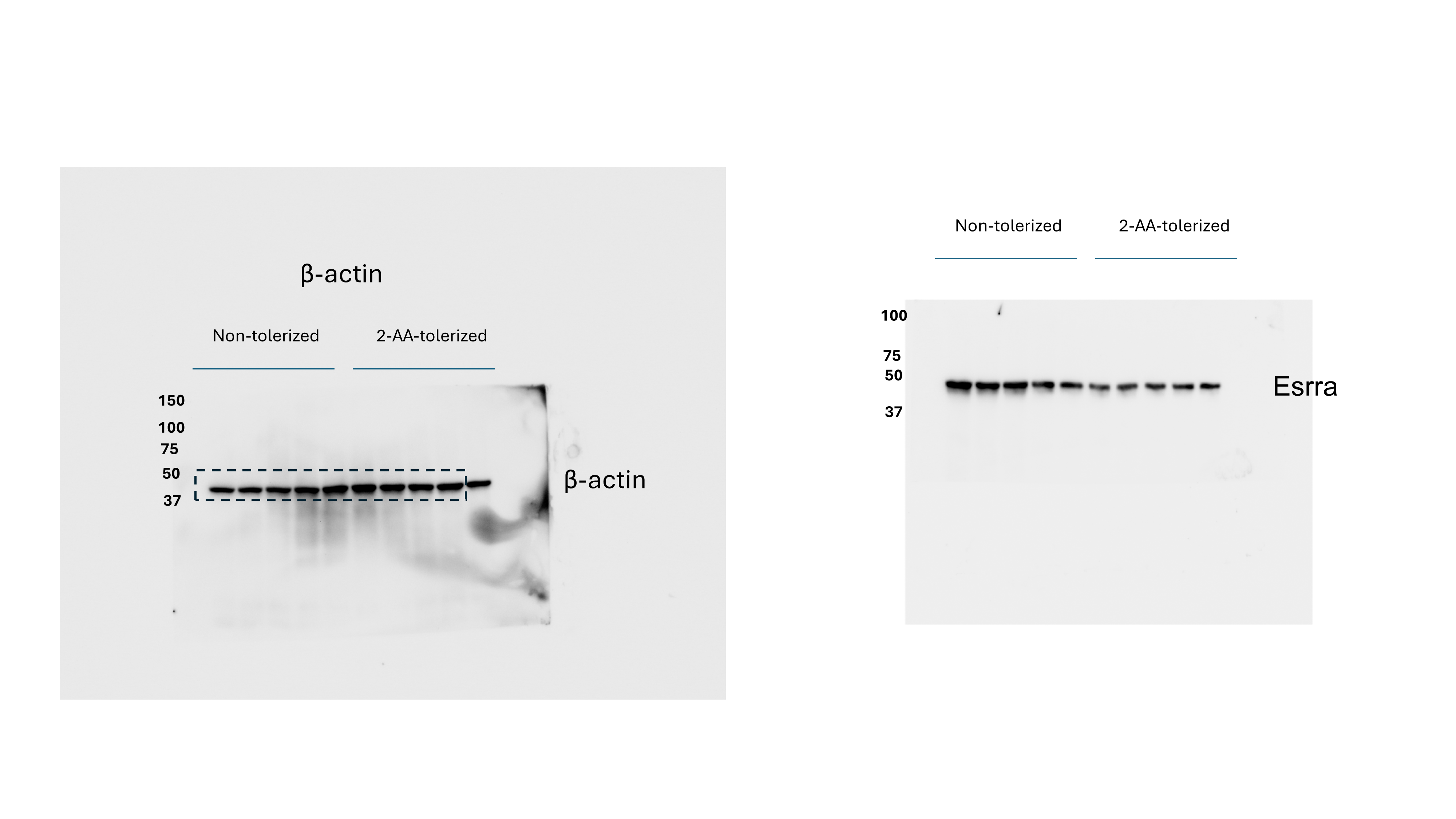

Supplement: Figure 3—source data 3. [file elife-97568-fig3-data3.zip › Figure 3—source data 1.tif]

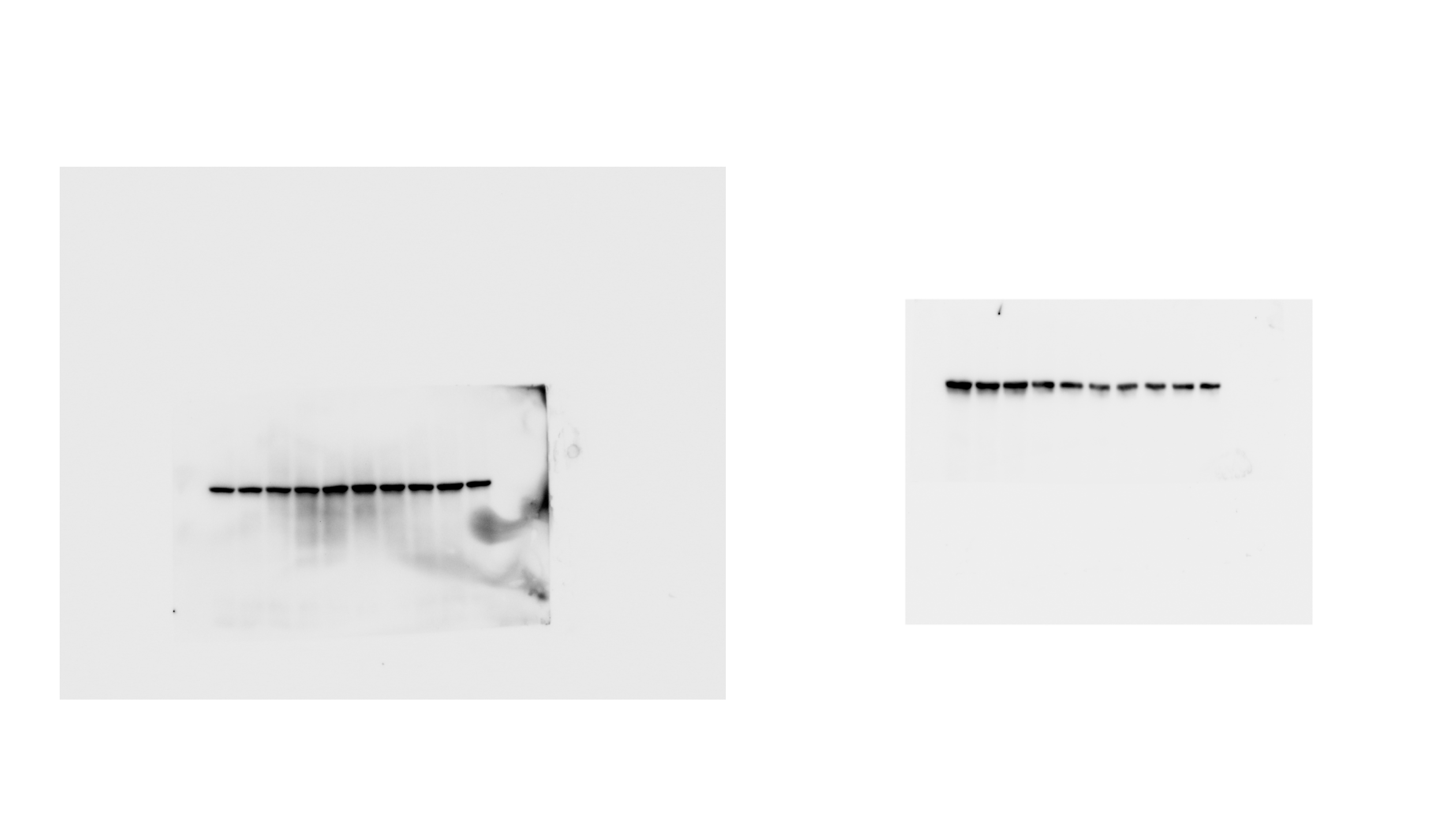

Supplement: Figure 3—source data 4. [file elife-97568-fig3-data4.zip › Figure 3—source data 2.tif]

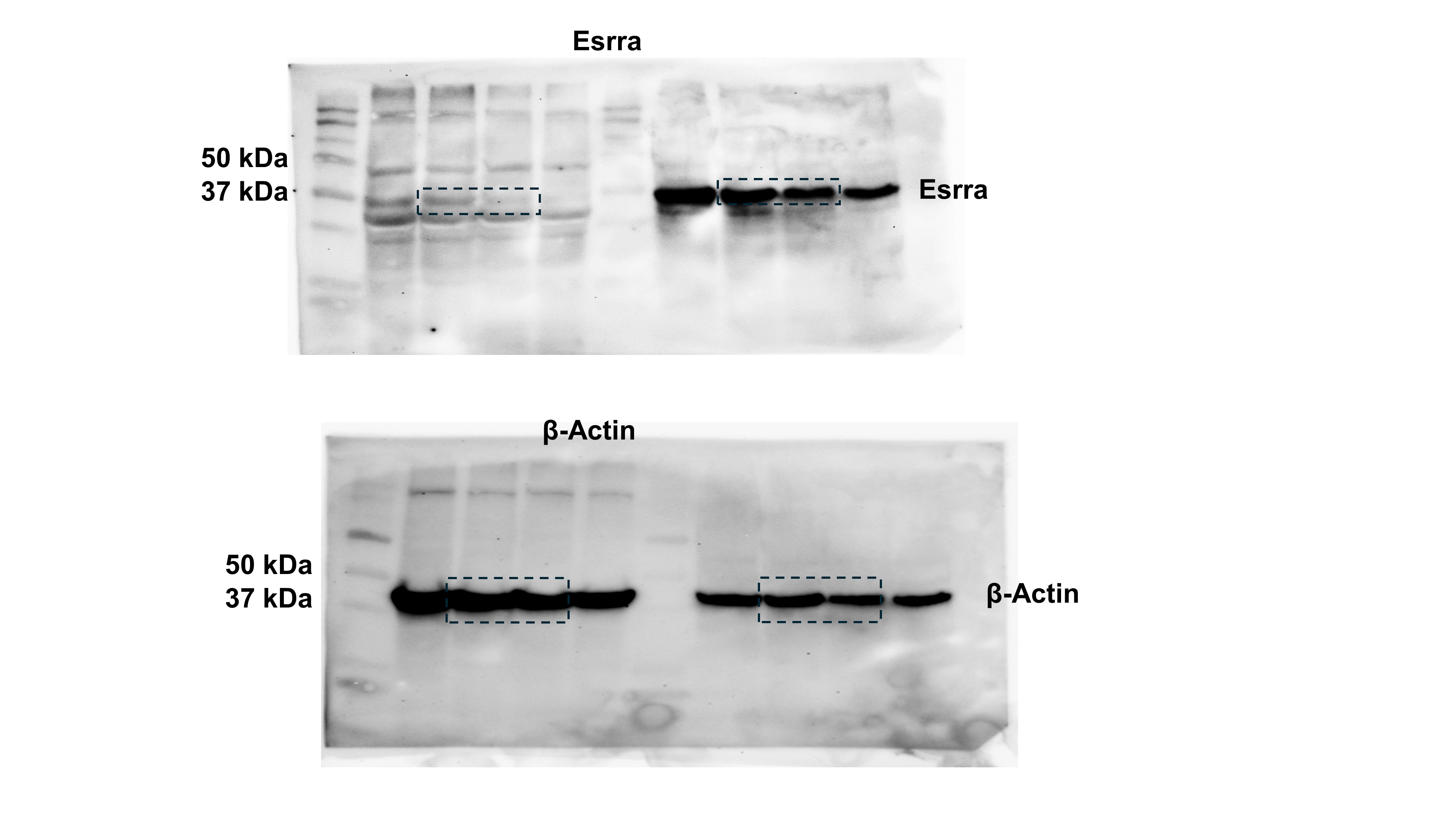

Supplement: Figure 3—source data 5. [file elife-97568-fig3-data5.zip › Figure 3—source data 3.tif]

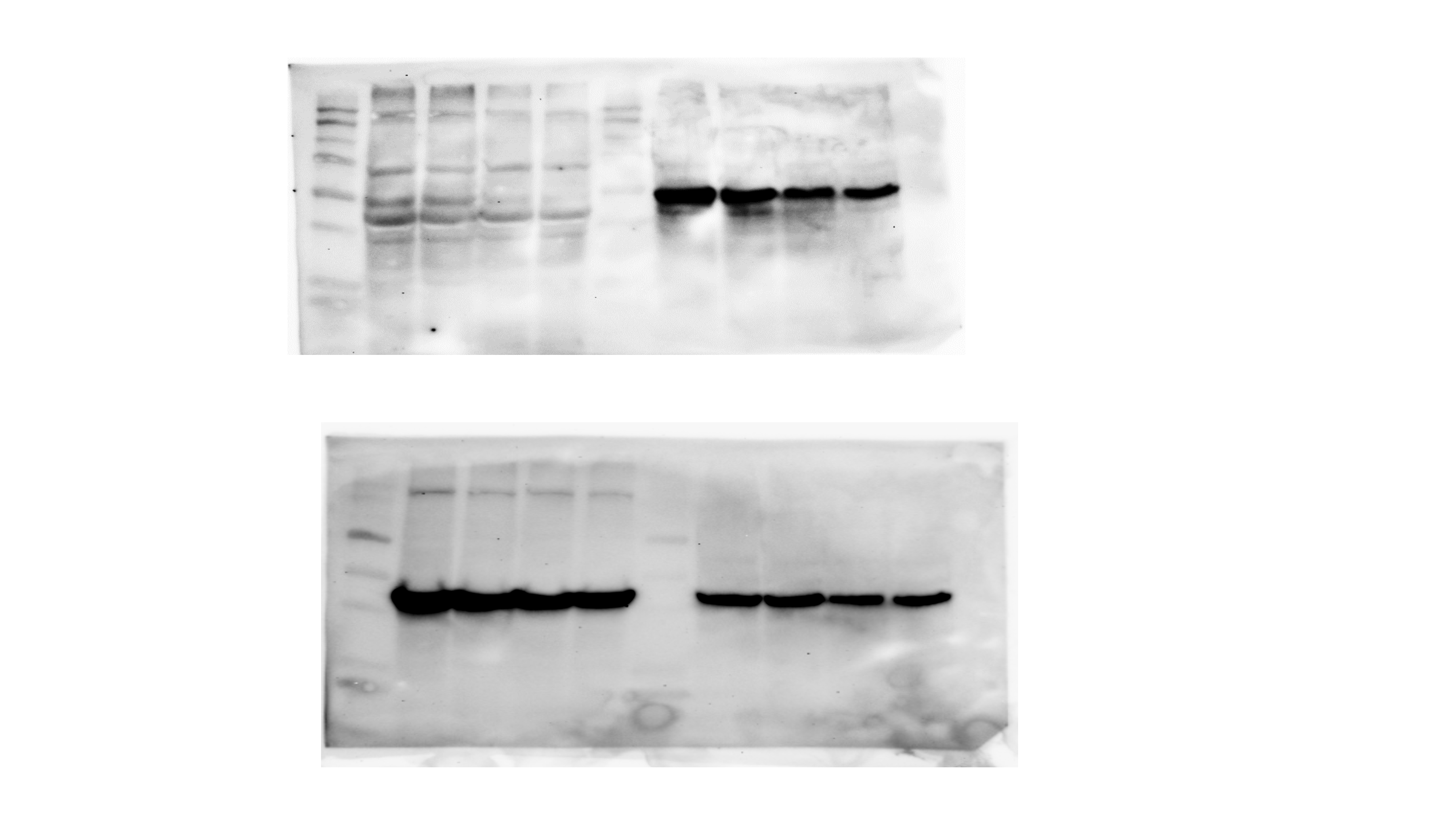

Supplement: Figure 3—source data 6. [file elife-97568-fig3-data6.zip › Figure 3—source data 4.tif]

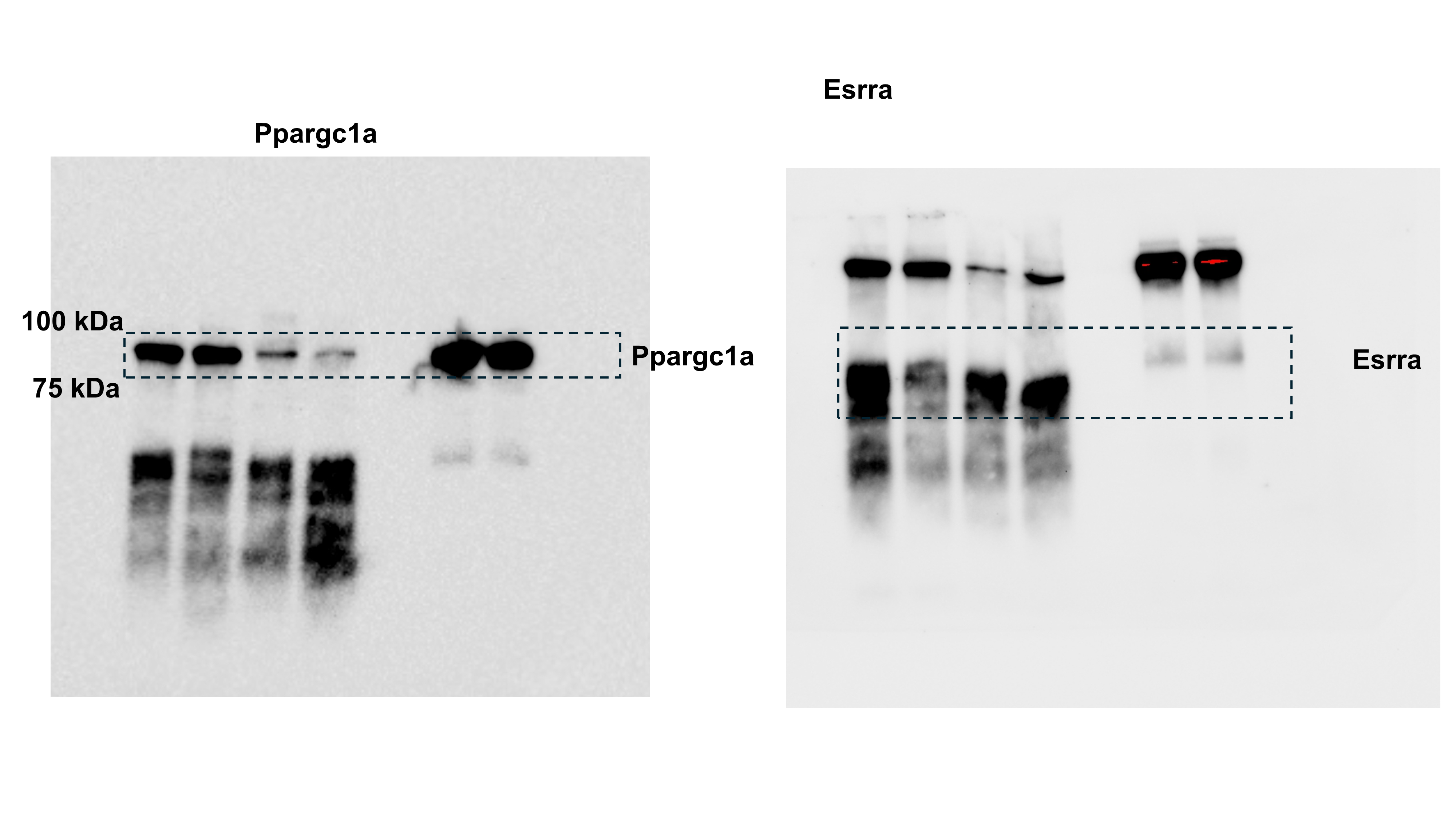

Supplement: Figure 3—source data 7. [file elife-97568-fig3-data7.zip › Figure 3—source data 5.tif]

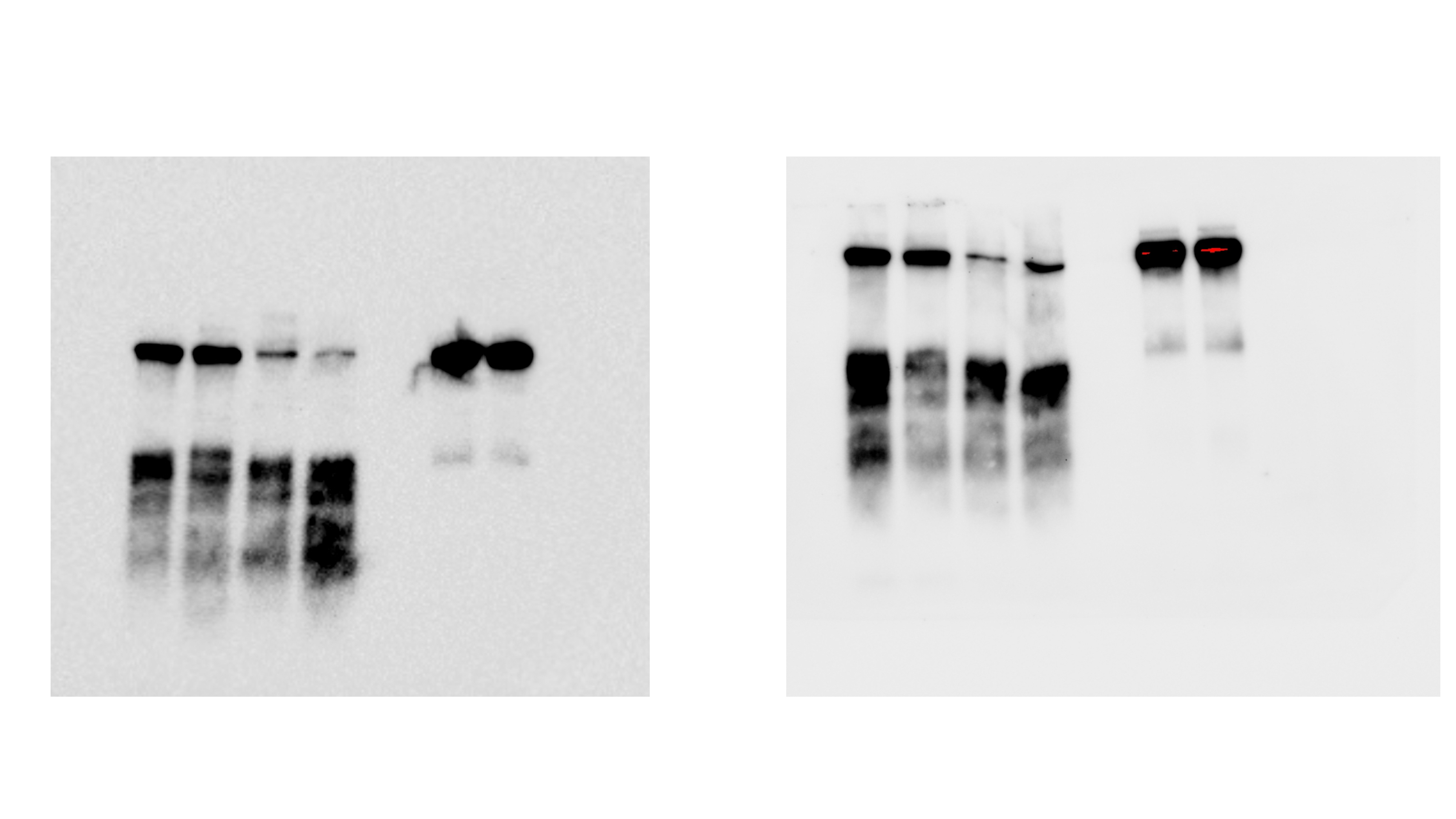

Supplement: Figure 3—source data 8. [file elife-97568-fig3-data8.zip › Figure 3—source data 6.tif]
